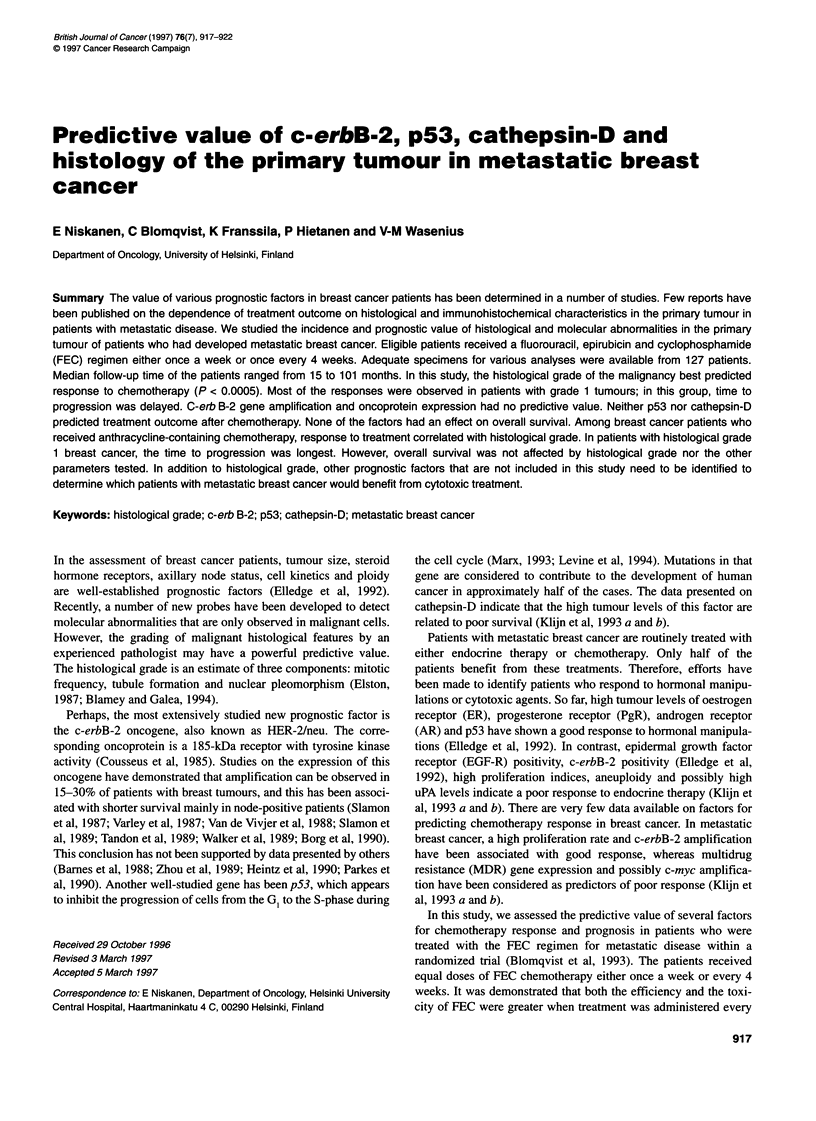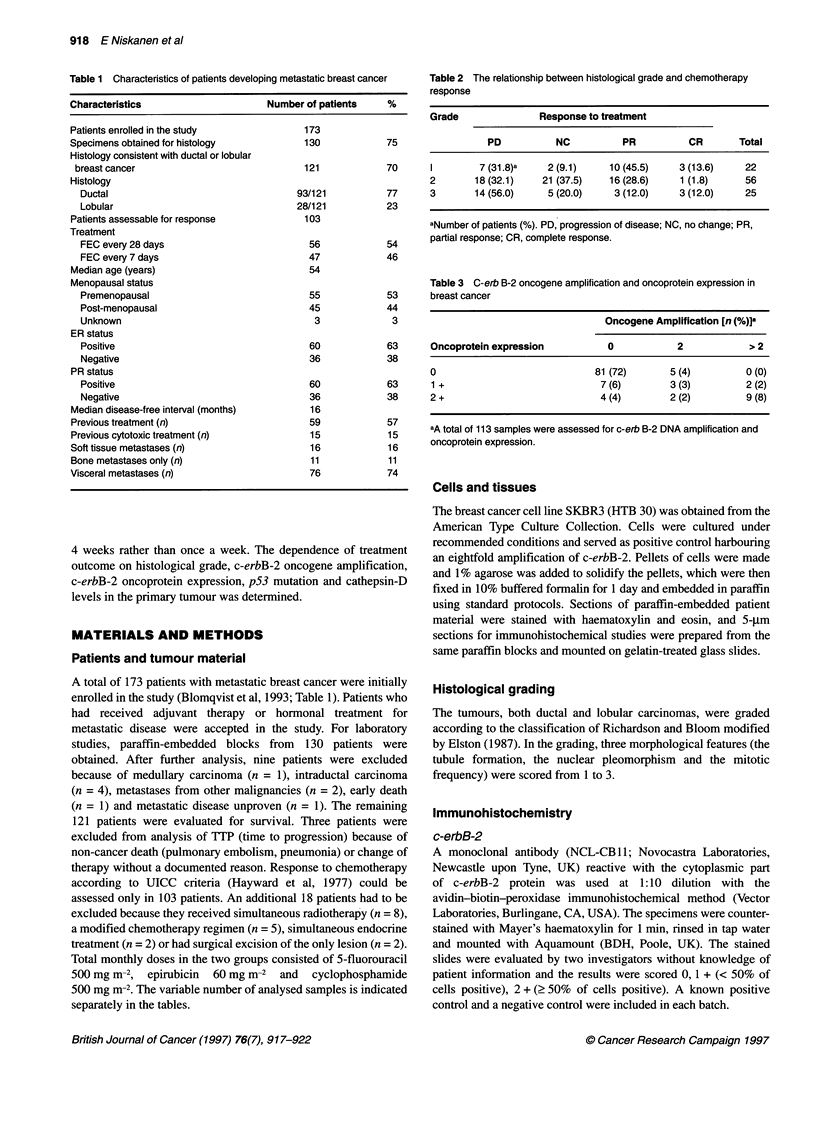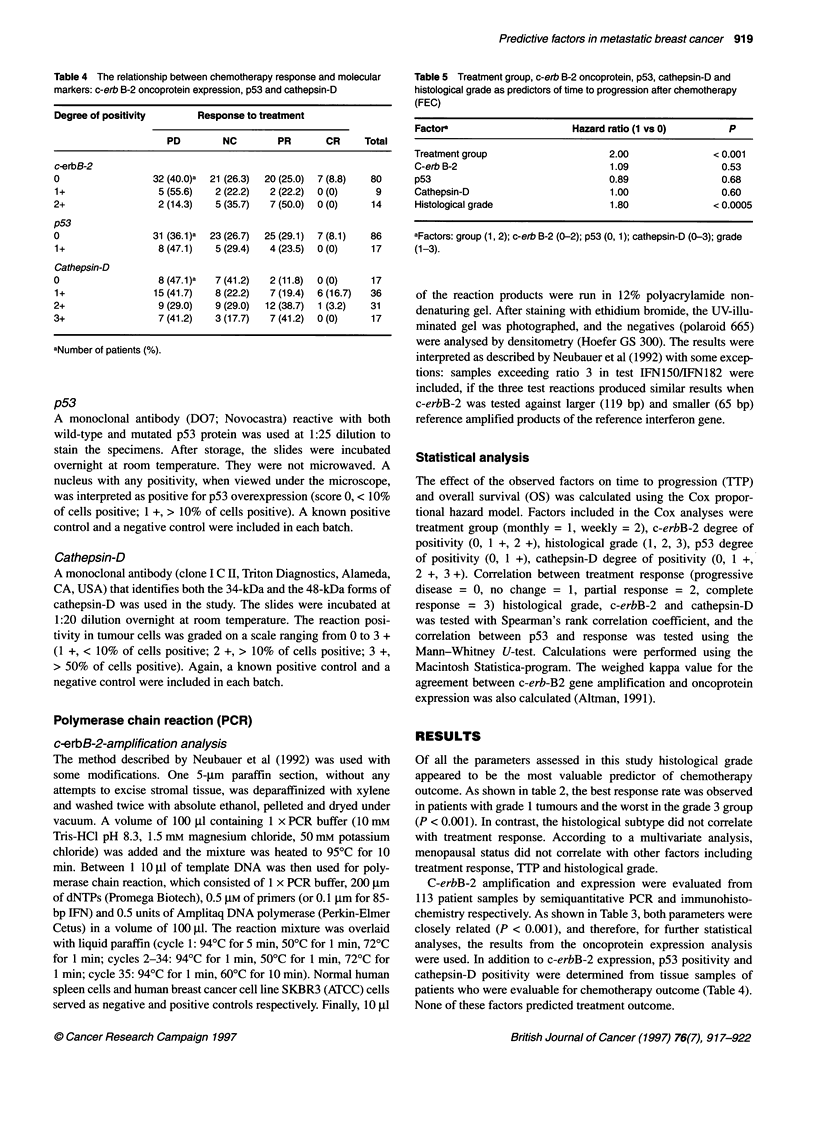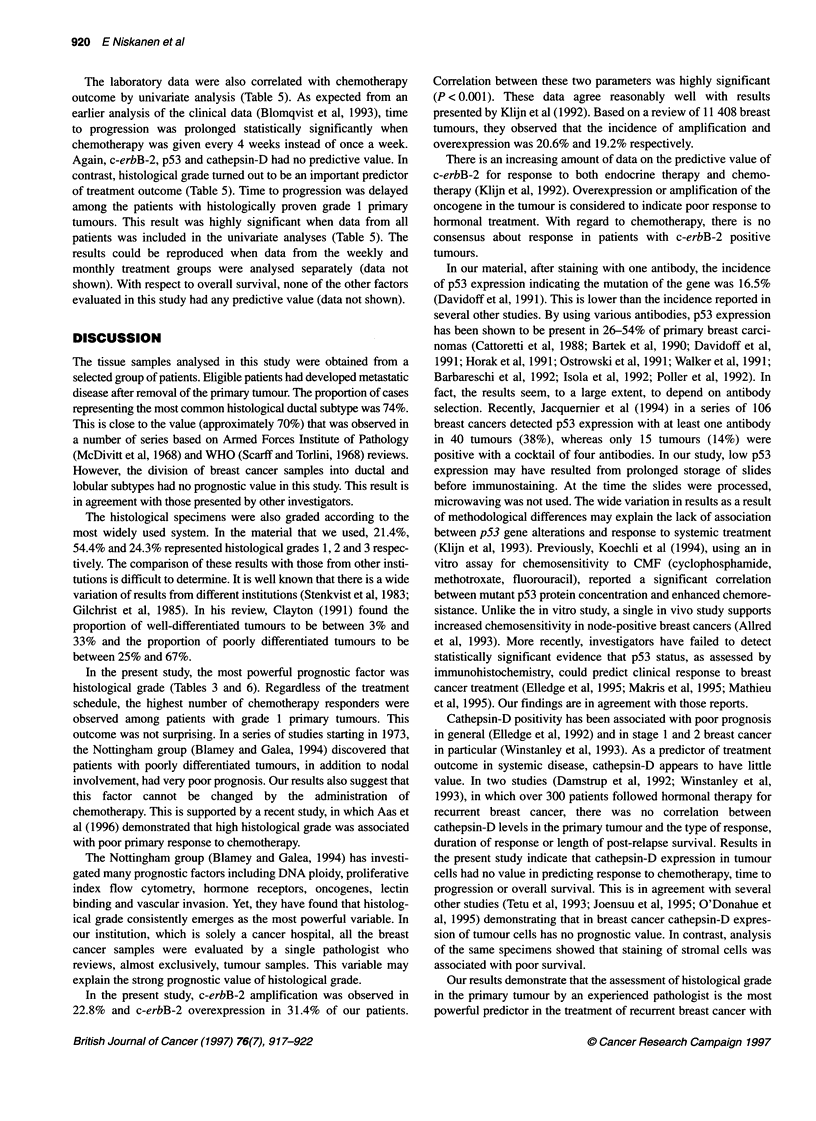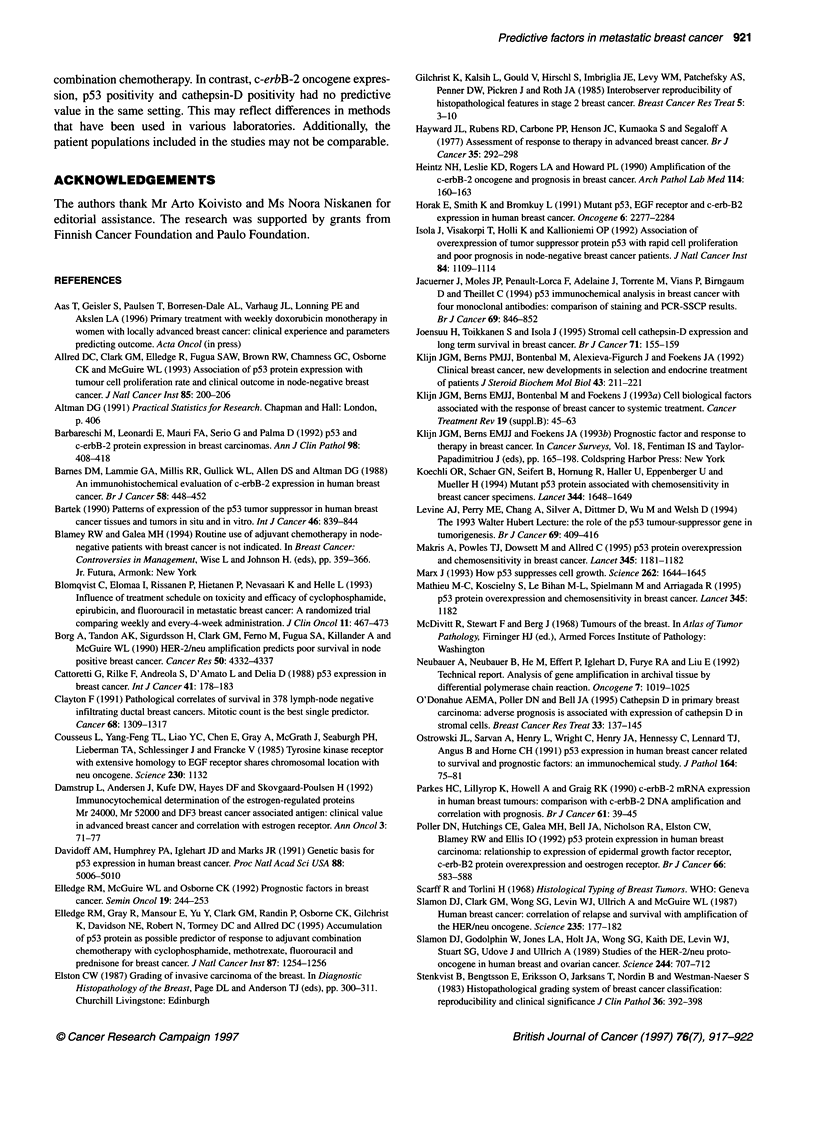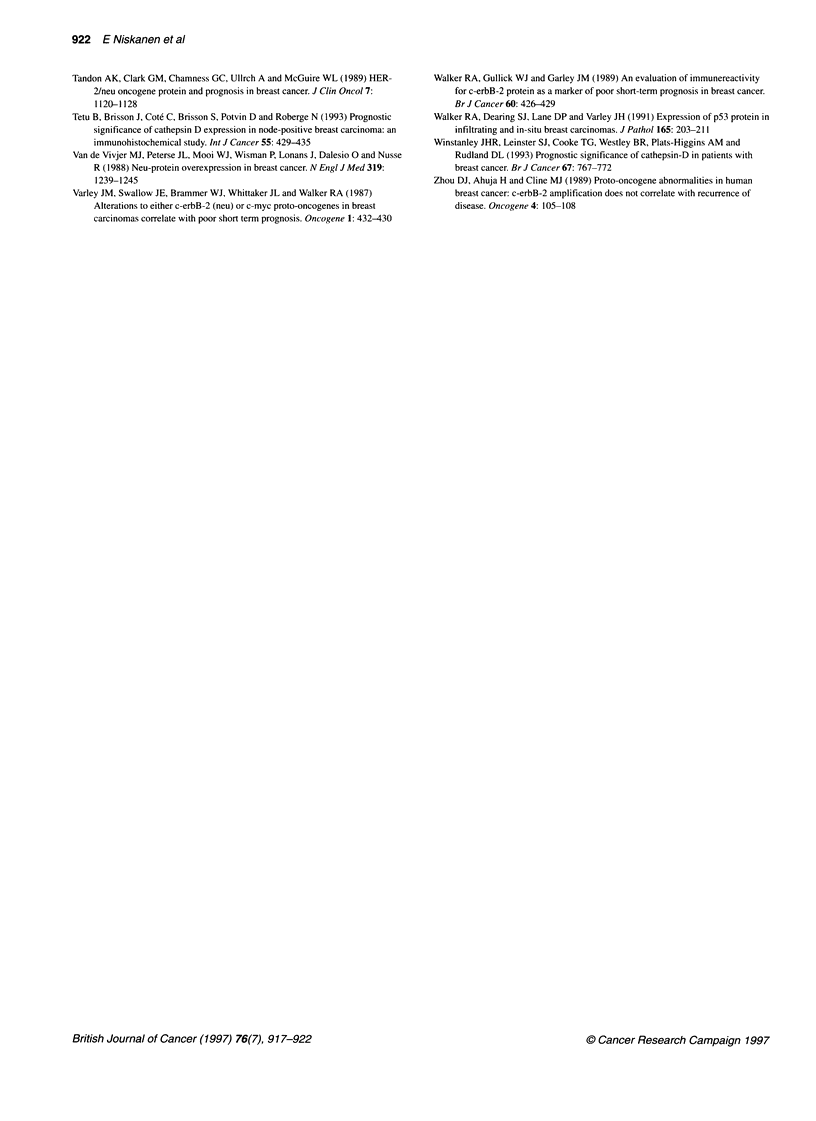# Predictive value of c-erbB-2, p53, cathepsin-D and histology of the primary tumour in metastatic breast cancer.

**DOI:** 10.1038/bjc.1997.484

**Published:** 1997

**Authors:** E. Niskanen, C. Blomqvist, K. Franssila, P. Hietanen, V. M. Wasenius

**Affiliations:** Department of Oncology, University of Helsinki, Finland.

## Abstract

The value of various prognostic factors in breast cancer patients has been determined in a number of studies. Few reports have been published on the dependence of treatment outcome on histological and immunohistochemical characteristics in the primary tumour in patients with metastatic disease. We studied the incidence and prognostic value of histological and molecular abnormalities in the primary tumour of patients who had developed metastatic breast cancer. Eligible patients received a fluorouracil, epirubicin and cyclophosphamide (FEC) regimen either once a week or once every 4 weeks. Adequate specimens for various analyses were available from 127 patients. Median follow-up time of the patients ranged from 15 to 101 months. In this study, the histological grade of the malignancy best predicted response to chemotherapy (P < 0.0005). Most of the responses were observed in patients with grade 1 tumours; in this group, time to progression was delayed. C-erb B-2 gene amplification and oncoprotein expression had no predictive value. Neither p53 nor cathepsin-D predicted treatment outcome after chemotherapy. None of the factors had an effect on overall survival. Among breast cancer patients who received anthracycline-containing chemotherapy, response to treatment correlated with histological grade. In patients with histological grade 1 breast cancer, the time to progression was longest. However, overall survival was not affected by histological grade nor the other parameters tested. In addition to histological grade, other prognostic factors that are not included in this study need to be identified to determine which patients with metastatic breast cancer would benefit from cytotoxic treatment.


British Journal of Cancer (1997) 76(7), 917–922
© 1997 Cancer Research Campaign

### Predictive value of c-*erb*B-2, p53, cathepsin-D and histology of the primary tumour in metastatic breast cancer

**E Niskanen, C Blomqvist, K Franssila, P Hietanen and V-M Wasenius**

Department of Oncology, University of Helsinki, Finland

**Summary** The value of various prognostic factors in breast cancer patients has been determined in a number of studies. Few reports have been published on the dependence of treatment outcome on histological and immunohistochemical characteristics in the primary tumour in patients with metastatic disease. We studied the incidence and prognostic value of histological and molecular abnormalities in the primary tumour of patients who had developed metastatic breast cancer. Eligible patients received a fluorouracil, epirubicin and cyclophosphamide (FEC) regimen either once a week or once every 4 weeks. Adequate specimens for various analyses were available from 127 patients. Median follow-up time of the patients ranged from 15 to 101 months. In this study, the histological grade of the malignancy best predicted response to chemotherapy ($P < 0.0005$). Most of the responses were observed in patients with grade 1 tumours; in this group, time to progression was delayed. C-*erb* B-2 gene amplification and oncoprotein expression had no predictive value. Neither p53 nor cathepsin-D predicted treatment outcome after chemotherapy. None of the factors had an effect on overall survival. Among breast cancer patients who received anthracycline-containing chemotherapy, response to treatment correlated with histological grade. In patients with histological grade 1 breast cancer, the time to progression was longest. However, overall survival was not affected by histological grade nor the other parameters tested. In addition to histological grade, other prognostic factors that are not included in this study need to be identified to determine which patients with metastatic breast cancer would benefit from cytotoxic treatment.

**Keywords:** histological grade; c-*erb* B-2; p53; cathepsin-D; metastatic breast cancer

In the assessment of breast cancer patients, tumour size, steroid hormone receptors, axillary node status, cell kinetics and ploidy are well-established prognostic factors (Elledge et al, 1992). Recently, a number of new probes have been developed to detect molecular abnormalities that are only observed in malignant cells. However, the grading of malignant histological features by an experienced pathologist may have a powerful predictive value. The histological grade is an estimate of three components: mitotic frequency, tubule formation and nuclear pleomorphism (Elston, 1987; Blamey and Galea, 1994).

Perhaps, the most extensively studied new prognostic factor is the c-*erb*B-2 oncogene, also known as HER-2/neu. The corresponding oncoprotein is a 185-kDa receptor with tyrosine kinase activity (Cousseus et al, 1985). Studies on the expression of this oncogene have demonstrated that amplification can be observed in 15–30% of patients with breast tumours, and this has been associated with shorter survival mainly in node-positive patients (Slamon et al, 1987; Varley et al, 1987; Van de Vivjer et al, 1988; Slamon et al, 1989; Tandon et al, 1989; Walker et al, 1989; Borg et al, 1990). This conclusion has not been supported by data presented by others (Barnes et al, 1988; Zhou et al, 1989; Heintz et al, 1990; Parkes et al, 1990). Another well-studied gene has been *p53*, which appears to inhibit the progression of cells from the $G_1$ to the S-phase during the cell cycle (Marx, 1993; Levine et al, 1994). Mutations in that gene are considered to contribute to the development of human cancer in approximately half of the cases. The data presented on cathepsin-D indicate that the high tumour levels of this factor are related to poor survival (Klijn et al, 1993 *a* and *b*).

Patients with metastatic breast cancer are routinely treated with either endocrine therapy or chemotherapy. Only half of the patients benefit from these treatments. Therefore, efforts have been made to identify patients who respond to hormonal manipulations or cytotoxic agents. So far, high tumour levels of oestrogen receptor (ER), progesterone receptor (PgR), androgen receptor (AR) and p53 have shown a good response to hormonal manipulations (Elledge et al, 1992). In contrast, epidermal growth factor receptor (EGF-R) positivity, c-*erb*B-2 positivity (Elledge et al, 1992), high proliferation indices, aneuploidy and possibly high uPA levels indicate a poor response to endocrine therapy (Klijn et al, 1993 *a* and *b*). There are very few data available on factors for predicting chemotherapy response in breast cancer. In metastatic breast cancer, a high proliferation rate and c-*erb*B-2 amplification have been associated with good response, whereas multidrug resistance (MDR) gene expression and possibly c-*myc* amplification have been considered as predictors of poor response (Klijn et al, 1993 *a* and *b*).

In this study, we assessed the predictive value of several factors for chemotherapy response and prognosis in patients who were treated with the FEC regimen for metastatic disease within a randomized trial (Blomqvist et al, 1993). The patients received equal doses of FEC chemotherapy either once a week or every 4 weeks. It was demonstrated that both the efficiency and the toxicity of FEC were greater when treatment was administered every

*Received 29 October 1996*
*Revised 3 March 1997*
*Accepted 5 March 1997*

*Correspondence to:* E Niskanen, Department of Oncology, Helsinki University Central Hospital, Haartmaninkatu 4 C, 00290 Helsinki, Finland

**Table 1** Characteristics of patients developing metastatic breast cancer

| Characteristics | Number of patients | % |
|---|---|---|
| Patients enrolled in the study | 173 | |
| Specimens obtained for histology | 130 | 75 |
| Histology consistent with ductal or lobular breast cancer | 121 | 70 |
| Histology | | |
| Ductal | 93/121 | 77 |
| Lobular | 28/121 | 23 |
| Patients assessable for response | 103 | |
| Treatment | | |
| FEC every 28 days | 56 | 54 |
| FEC every 7 days | 47 | 46 |
| Median age (years) | 54 | |
| Menopausal status | | |
| Premenopausal | 55 | 53 |
| Post-menopausal | 45 | 44 |
| Unknown | 3 | 3 |
| ER status | | |
| Positive | 60 | 63 |
| Negative | 36 | 38 |
| PR status | | |
| Positive | 60 | 63 |
| Negative | 36 | 38 |
| Median disease-free interval (months) | 16 | |
| Previous treatment (*n*) | 59 | 57 |
| Previous cytotoxic treatment (*n*) | 15 | 15 |
| Soft tissue metastases (*n*) | 16 | 16 |
| Bone metastases only (*n*) | 11 | 11 |
| Visceral metastases (*n*) | 76 | 74 |

4 weeks rather than once a week. The dependence of treatment outcome on histological grade, c-*erb*B-2 oncogene amplification, c-*erb*B-2 oncoprotein expression, *p53* mutation and cathepsin-D levels in the primary tumour was determined.

### MATERIALS AND METHODS

### Patients and tumour material

A total of 173 patients with metastatic breast cancer were initially enrolled in the study (Blomqvist et al, 1993; Table 1). Patients who had received adjuvant therapy or hormonal treatment for metastatic disease were accepted in the study. For laboratory studies, paraffin-embedded blocks from 130 patients were obtained. After further analysis, nine patients were excluded because of medullary carcinoma ($n$ = 1), intraductal carcinoma ($n$ = 4), metastases from other malignancies ($n$ = 2), early death ($n$ = 1) and metastatic disease unproven ($n$ = 1). The remaining 121 patients were evaluated for survival. Three patients were excluded from analysis of TTP (time to progression) because of non-cancer death (pulmonary embolism, pneumonia) or change of therapy without a documented reason. Response to chemotherapy according to UICC criteria (Hayward et al, 1977) could be assessed only in 103 patients. An additional 18 patients had to be excluded because they received simultaneous radiotherapy ($n$ = 8), a modified chemotherapy regimen ($n$ = 5), simultaneous endocrine treatment ($n$ = 2) or had surgical excision of the only lesion ($n$ = 2). Total monthly doses in the two groups consisted of 5-fluorouracil 500 mg $m^{-2}$, epirubicin 60 mg $m^{-2}$ and cyclophosphamide 500 mg $m^{-2}$. The variable number of analysed samples is indicated separately in the tables.

**Table 2** The relationship between histological grade and chemotherapy response

| Grade | Response to treatment | | | | |
|---|---|---|---|---|---|
| | PD | NC | PR | CR | Total |
| I | 7 (31.8)[a] | 2 (9.1) | 10 (45.5) | 3 (13.6) | 22 |
| 2 | 18 (32.1) | 21 (37.5) | 16 (28.6) | 1 (1.8) | 56 |
| 3 | 14 (56.0) | 5 (20.0) | 3 (12.0) | 3 (12.0) | 25 |

[a]Number of patients (%). PD, progression of disease; NC, no change; PR, partial response; CR, complete response.

**Table 3** C-*erb* B-2 oncogene amplification and oncoprotein expression in breast cancer

| | Oncogene Amplification [*n* (%)][a] | | |
|---|---|---|---|
| Oncoprotein expression | 0 | 2 | >2 |
| 0 | 81 (72) | 5 (4) | 0 (0) |
| 1 + | 7 (6) | 3 (3) | 2 (2) |
| 2 + | 4 (4) | 2 (2) | 9 (8) |

[a]A total of 113 samples were assessed for c-*erb* B-2 DNA amplification and oncoprotein expression.

### Cells and tissues

The breast cancer cell line SKBR3 (HTB 30) was obtained from the American Type Culture Collection. Cells were cultured under recommended conditions and served as positive control harbouring an eightfold amplification of c-*erb*B-2. Pellets of cells were made and 1% agarose was added to solidify the pellets, which were then fixed in 10% buffered formalin for 1 day and embedded in paraffin using standard protocols. Sections of paraffin-embedded patient material were stained with haematoxylin and eosin, and 5-μm sections for immunohistochemical studies were prepared from the same paraffin blocks and mounted on gelatin-treated glass slides.

### Histological grading

The tumours, both ductal and lobular carcinomas, were graded according to the classification of Richardson and Bloom modified by Elston (1987). In the grading, three morphological features (the tubule formation, the nuclear pleomorphism and the mitotic frequency) were scored from 1 to 3.

### Immunohistochemistry

### *c-erbB-2*

A monoclonal antibody (NCL-CB11; Novocastra Laboratories, Newcastle upon Tyne, UK) reactive with the cytoplasmic part of c-*erb*B-2 protein was used at 1:10 dilution with the avidin–biotin–peroxidase immunohistochemical method (Vector Laboratories, Burlingane, CA, USA). The specimens were counter-stained with Mayer's haematoxylin for 1 min, rinsed in tap water and mounted with Aquamount (BDH, Poole, UK). The stained slides were evaluated by two investigators without knowledge of patient information and the results were scored 0, 1 + (< 50% of cells positive), 2 + (≥ 50% of cells positive). A known positive control and a negative control were included in each batch.

© Cancer Research Campaign 1997

Table 4 The relationship between chemotherapy response and molecular markers: c-*erb* B-2 oncoprotein expression, p53 and cathepsin-D

| Degree of positivity | Response to treatment | | | | |
|---|---|---|---|---|---|
| | PD | NC | PR | CR | Total |
| *c-*erb*B-2* | | | | | |
| 0 | 32 (40.0)[a] | 21 (26.3) | 20 (25.0) | 7 (8.8) | 80 |
| 1+ | 5 (55.6) | 2 (22.2) | 2 (22.2) | 0 (0) | 9 |
| 2+ | 2 (14.3) | 5 (35.7) | 7 (50.0) | 0 (0) | 14 |
| *p53* | | | | | |
| 0 | 31 (36.1)[a] | 23 (26.7) | 25 (29.1) | 7 (8.1) | 86 |
| 1+ | 8 (47.1) | 5 (29.4) | 4 (23.5) | 0 (0) | 17 |
| *Cathepsin-D* | | | | | |
| 0 | 8 (47.1)[a] | 7 (41.2) | 2 (11.8) | 0 (0) | 17 |
| 1+ | 15 (41.7) | 8 (22.2) | 7 (19.4) | 6 (16.7) | 36 |
| 2+ | 9 (29.0) | 9 (29.0) | 12 (38.7) | 1 (3.2) | 31 |
| 3+ | 7 (41.2) | 3 (17.7) | 7 (41.2) | 0 (0) | 17 |

[a]Number of patients (%).

### *p53*

A monoclonal antibody (DO7; Novocastra) reactive with both wild-type and mutated p53 protein was used at 1:25 dilution to stain the specimens. After storage, the slides were incubated overnight at room temperature. They were not microwaved. A nucleus with any positivity, when viewed under the microscope, was interpreted as positive for p53 overexpression (score 0, < 10% of cells positive; 1 +, > 10% of cells positive). A known positive control and a negative control were included in each batch.

### *Cathepsin-D*

A monoclonal antibody (clone I C II, Triton Diagnostics, Alameda, CA, USA) that identifies both the 34-kDa and the 48-kDa forms of cathepsin-D was used in the study. The slides were incubated at 1:20 dilution overnight at room temperature. The reaction positivity in tumour cells was graded on a scale ranging from 0 to 3 + (1 +, < 10% of cells positive; 2 +, > 10% of cells positive; 3 +, > 50% of cells positive). Again, a known positive control and a negative control were included in each batch.

### Polymerase chain reaction (PCR)

### *c-*erb*B-2-amplification analysis*

The method described by Neubauer et al (1992) was used with some modifications. One 5-μm paraffin section, without any attempts to excise stromal tissue, was deparaffinized with xylene and washed twice with absolute ethanol, pelleted and dryed under vacuum. A volume of 100 μl containing 1 × PCR buffer (10 mM Tris-HCl pH 8.3, 1.5 mM magnesium chloride, 50 mM potassium chloride) was added and the mixture was heated to 95°C for 10 min. Between 1 10 μl of template DNA was then used for polymerase chain reaction, which consisted of 1 × PCR buffer, 200 μm of dNTPs (Promega Biotech), 0.5 μM of primers (or 0.1 μm for 85-bp IFN) and 0.5 units of Amplitaq DNA polymerase (Perkin-Elmer Cetus) in a volume of 100 μl. The reaction mixture was overlaid with liquid paraffin (cycle 1: 94°C for 5 min, 50°C for 1 min, 72°C for 1 min; cycles 2–34: 94°C for 1 min, 50°C for 1 min, 72°C for 1 min; cycle 35: 94°C for 1 min, 60°C for 10 min). Normal human spleen cells and human breast cancer cell line SKBR3 (ATCC) cells served as negative and positive controls respectively. Finally, 10 μl of the reaction products were run in 12% polyacrylamide non-denaturing gel. After staining with ethidium bromide, the UV-illuminated gel was photographed, and the negatives (polaroid 665) were analysed by densitometry (Hoefer GS 300). The results were interpreted as described by Neubauer et al (1992) with some exceptions: samples exceeding ratio 3 in test IFN150/IFN182 were included, if the three test reactions produced similar results when c-*erb*B-2 was tested against larger (119 bp) and smaller (65 bp) reference amplified products of the reference interferon gene.

Table 5 Treatment group, c-*erb* B-2 oncoprotein, p53, cathepsin-D and histological grade as predictors of time to progression after chemotherapy (FEC)

| Factor[a] | Hazard ratio (1 vs 0) | P |
|---|---|---|
| Treatment group | 2.00 | < 0.001 |
| C-*erb* B-2 | 1.09 | 0.53 |
| p53 | 0.89 | 0.68 |
| Cathepsin-D | 1.00 | 0.60 |
| Histological grade | 1.80 | < 0.0005 |

[a]Factors: group (1, 2); c-*erb* B-2 (0–2); p53 (0, 1); cathepsin-D (0–3); grade (1–3).

### Statistical analysis

The effect of the observed factors on time to progression (TTP) and overall survival (OS) was calculated using the Cox proportional hazard model. Factors included in the Cox analyses were treatment group (monthly = 1, weekly = 2), c-*erb*B-2 degree of positivity (0, 1 +, 2 +), histological grade (1, 2, 3), p53 degree of positivity (0, 1 +), cathepsin-D degree of positivity (0, 1 +, 2 +, 3 +). Correlation between treatment response (progressive disease = 0, no change = 1, partial response = 2, complete response = 3) histological grade, c-*erb*B-2 and cathepsin-D was tested with Spearman's rank correlation coefficient, and the correlation between p53 and response was tested using the Mann–Whitney *U*-test. Calculations were performed using the Macintosh Statistica-program. The weighed kappa value for the agreement between c-*erb*-B2 gene amplification and oncoprotein expression was also calculated (Altman, 1991).

### RESULTS

Of all the parameters assessed in this study histological grade appeared to be the most valuable predictor of chemotherapy outcome. As shown in table 2, the best response rate was observed in patients with grade 1 tumours and the worst in the grade 3 group ($P < 0.001$). In contrast, the histological subtype did not correlate with treatment response. According to a multivariate analysis, menopausal status did not correlate with other factors including treatment response, TTP and histological grade.

C-*erb*B-2 amplification and expression were evaluated from 113 patient samples by semiquantitative PCR and immunohistochemistry respectively. As shown in Table 3, both parameters were closely related ($P < 0.001$), and therefore, for further statistical analyses, the results from the oncoprotein expression analysis were used. In addition to c-*erb*B-2 expression, p53 positivity and cathepsin-D positivity were determined from tissue samples of patients who were evaluable for chemotherapy outcome (Table 4). None of these factors predicted treatment outcome.

© Cancer Research Campaign 1997

The laboratory data were also correlated with chemotherapy outcome by univariate analysis (Table 5). As expected from an earlier analysis of the clinical data (Blomqvist et al, 1993), time to progression was prolonged statistically significantly when chemotherapy was given every 4 weeks instead of once a week. Again, c-*erb*B-2, p53 and cathepsin-D had no predictive value. In contrast, histological grade turned out to be an important predictor of treatment outcome (Table 5). Time to progression was delayed among the patients with histologically proven grade 1 primary tumours. This result was highly significant when data from all patients was included in the univariate analyses (Table 5). The results could be reproduced when data from the weekly and monthly treatment groups were analysed separately (data not shown). With respect to overall survival, none of the other factors evaluated in this study had any predictive value (data not shown).

### DISCUSSION

The tissue samples analysed in this study were obtained from a selected group of patients. Eligible patients had developed metastatic disease after removal of the primary tumour. The proportion of cases representing the most common histological ductal subtype was 74%. This is close to the value (approximately 70%) that was observed in a number of series based on Armed Forces Institute of Pathology (McDivitt et al, 1968) and WHO (Scarff and Torlini, 1968) reviews. However, the division of breast cancer samples into ductal and lobular subtypes had no prognostic value in this study. This result is in agreement with those presented by other investigators.

The histological specimens were also graded according to the most widely used system. In the material that we used, 21.4%, 54.4% and 24.3% represented histological grades 1, 2 and 3 respectively. The comparison of these results with those from other institutions is difficult to determine. It is well known that there is a wide variation of results from different institutions (Stenkvist et al, 1983; Gilchrist et al, 1985). In his review, Clayton (1991) found the proportion of well-differentiated tumours to be between 3% and 33% and the proportion of poorly differentiated tumours to be between 25% and 67%.

In the present study, the most powerful prognostic factor was histological grade (Tables 3 and 6). Regardless of the treatment schedule, the highest number of chemotherapy responders were observed among patients with grade 1 primary tumours. This outcome was not surprising. In a series of studies starting in 1973, the Nottingham group (Blamey and Galea, 1994) discovered that patients with poorly differentiated tumours, in addition to nodal involvement, had very poor prognosis. Our results also suggest that this factor cannot be changed by the administration of chemotherapy. This is supported by a recent study, in which Aas et al (1996) demonstrated that high histological grade was associated with poor primary response to chemotherapy.

The Nottingham group (Blamey and Galea, 1994) has investigated many prognostic factors including DNA ploidy, proliferative index flow cytometry, hormone receptors, oncogenes, lectin binding and vascular invasion. Yet, they have found that histological grade consistently emerges as the most powerful variable. In our institution, which is solely a cancer hospital, all the breast cancer samples were evaluated by a single pathologist who reviews, almost exclusively, tumour samples. This variable may explain the strong prognostic value of histological grade.

In the present study, c-*erb*B-2 amplification was observed in 22.8% and c-*erb*B-2 overexpression in 31.4% of our patients. Correlation between these two parameters was highly significant ($P < 0.001$). These data agree reasonably well with results presented by Klijn et al (1992). Based on a review of 11 408 breast tumours, they observed that the incidence of amplification and overexpression was 20.6% and 19.2% respectively.

There is an increasing amount of data on the predictive value of c-*erb*B-2 for response to both endocrine therapy and chemotherapy (Klijn et al, 1992). Overexpression or amplification of the oncogene in the tumour is considered to indicate poor response to hormonal treatment. With regard to chemotherapy, there is no consensus about response in patients with c-*erb*B-2 positive tumours.

In our material, after staining with one antibody, the incidence of p53 expression indicating the mutation of the gene was 16.5% (Davidoff et al, 1991). This is lower than the incidence reported in several other studies. By using various antibodies, p53 expression has been shown to be present in 26–54% of primary breast carcinomas (Cattoretti et al, 1988; Bartek et al, 1990; Davidoff et al, 1991; Horak et al, 1991; Ostrowski et al, 1991; Walker et al, 1991; Barbareschi et al, 1992; Isola et al, 1992; Poller et al, 1992). In fact, the results seem, to a large extent, to depend on antibody selection. Recently, Jacquemier et al (1994) in a series of 106 breast cancers detected p53 expression with at least one antibody in 40 tumours (38%), whereas only 15 tumours (14%) were positive with a cocktail of four antibodies. In our study, low p53 expression may have resulted from prolonged storage of slides before immunostaining. At the time the slides were processed, microwaving was not used. The wide variation in results as a result of methodological differences may explain the lack of association between *p53* gene alterations and response to systemic treatment (Klijn et al, 1993). Previously, Koechli et al (1994), using an in vitro assay for chemosensitivity to CMF (cyclophosphamide, methotroxate, fluorouracil), reported a significant correlation between mutant p53 protein concentration and enhanced chemoresistance. Unlike the in vitro study, a single in vivo study supports increased chemosensitivity in node-positive breast cancers (Allred et al, 1993). More recently, investigators have failed to detect statistically significant evidence that p53 status, as assessed by immunohistochemistry, could predict clinical response to breast cancer treatment (Elledge et al, 1995; Makris et al, 1995; Mathieu et al, 1995). Our findings are in agreement with those reports.

Cathepsin-D positivity has been associated with poor prognosis in general (Elledge et al, 1992) and in stage 1 and 2 breast cancer in particular (Winstanley et al, 1993). As a predictor of treatment outcome in systemic disease, cathepsin-D appears to have little value. In two studies (Damstrup et al, 1992; Winstanley et al, 1993), in which over 300 patients followed hormonal therapy for recurrent breast cancer, there was no correlation between cathepsin-D levels in the primary tumour and the type of response, duration of response or length of post-relapse survival. Results in the present study indicate that cathepsin-D expression in tumour cells had no value in predicting response to chemotherapy, time to progression or overall survival. This is in agreement with several other studies (Tetu et al, 1993; Joensuu et al, 1995; O'Donahue et al, 1995) demonstrating that in breast cancer cathepsin-D expression of tumour cells has no prognostic value. In contrast, analysis of the same specimens showed that staining of stromal cells was associated with poor survival.

Our results demonstrate that the assessment of histological grade in the primary tumour by an experienced pathologist is the most powerful predictor in the treatment of recurrent breast cancer with

© Cancer Research Campaign 1997

combination chemotherapy. In contrast, c-*erb*B-2 oncogene expression, p53 positivity and cathepsin-D positivity had no predictive value in the same setting. This may reflect differences in methods that have been used in various laboratories. Additionally, the patient populations included in the studies may not be comparable.

### ACKNOWLEDGEMENTS

The authors thank Mr Arto Koivisto and Ms Noora Niskanen for editorial assistance. The research was supported by grants from Finnish Cancer Foundation and Paulo Foundation.

### REFERENCES

Aas T, Geisler S, Paulsen T, Borresen-Dale AL, Varhaug JL, Lonning PE and Akslen LA (1996) Primary treatment with weekly doxorubicin monotherapy in women with locally advanced breast cancer: clinical experience and parameters predicting outcome. *Acta Oncol* (in press)

Allred DC, Clark GM, Elledge R, Fugua SAW, Brown RW, Chamness GC, Osborne CK and McGuire WL (1993) Association of p53 protein expression with tumour cell proliferation rate and clinical outcome in node-negative breast cancer. *J Natl Cancer Inst* **85**: 200–206

Altman DG (1991) *Practical Statistics for Research*. Chapman and Hall: London, p. 406

Barbareschi M, Leonardi E, Mauri FA, Serio G and Palma D (1992) p53 and c-erbB-2 protein expression in breast carcinomas. *Ann J Clin Pathol* **98**: 408–418

Barnes DM, Lammie GA, Millis RR, Gullick WL, Allen DS and Altman DG (1988) An immunohistochemical evaluation of c-erbB-2 expression in human breast cancer. *Br J Cancer* **58**: 448–452

Bartek (1990) Patterns of expression of the p53 tumor suppressor in human breast cancer tissues and tumors in situ and in vitro. *Int J Cancer* **46**: 839–844

Blamey RW and Galea MH (1994) Routine use of adjuvant chemotherapy in node-negative patients with breast cancer is not indicated. In *Breast Cancer: Controversies in Management*, Wise L and Johnson H. (eds), pp. 359–366. Jr. Futura, Armonk: New York

Blomqvist C, Elomaa I, Rissanen P, Hietanen P, Nevasaari K and Helle L (1993) Influence of treatment schedule on toxicity and efficacy of cyclophosphamide, epirubicin, and fluorouracil in metastatic breast cancer: A randomized trial comparing weekly and every-4-week administration. *J Clin Oncol* **11**: 467–473

Borg A, Tandon AK, Sigurdsson H, Clark GM, Ferno M, Fugua SA, Killander A and McGuire WL (1990) HER-2/neu amplification predicts poor survival in node positive breast cancer. *Cancer Res* **50**: 4332–4337

Cattoretti G, Rilke F, Andreola S, D'Amato L and Delia D (1988) p53 expression in breast cancer. *Int J Cancer* **41**: 178–183

Clayton F (1991) Pathological correlates of survival in 378 lymph-node negative infiltrating ductal breast cancers. Mitotic count is the best single predictor. *Cancer* **68**: 1309–1317

Cousseus L, Yang-Feng TL, Liao YC, Chen E, Gray A, McGrath J, Seaburgh PH, Lieberman TA, Schlessinger J and Francke V (1985) Tyrosine kinase receptor with extensive homology to EGF receptor shares chromosomal location with neu oncogene. *Science* **230**: 1132

Damstrup L, Andersen J, Kufe DW, Hayes DF and Skovgaard-Poulsen H (1992) Immunocytochemical determination of the estrogen-regulated proteins Mr 24000, Mr 52000 and DF3 breast cancer associated antigen: clinical value in advanced breast cancer and correlation with estrogen receptor. *Ann Oncol* **3**: 71–77

Davidoff AM, Humphrey PA, Iglehart JD and Marks JR (1991) Genetic basis for p53 expression in human breast cancer. *Proc Natl Acad Sci USA* **88**: 5006–5010

Elledge RM, McGuire WL and Osborne CK (1992) Prognostic factors in breast cancer. *Semin Oncol* **19**: 244–253

Elledge RM, Gray R, Mansour E, Yu Y, Clark GM, Randin P, Osborne CK, Gilchrist K, Davidson NE, Robert N, Tormey DC and Allred DC (1995) Accumulation of p53 protein as possible predictor of response to adjuvant combination chemotherapy with cyclophosphamide, methotrexate, fluorouracil and prednisone for breast cancer. *J Natl Cancer Inst* **87**: 1254–1256

Elston CW (1987) Grading of invasive carcinoma of the breast. In *Diagnostic Histopathology of the Breast*, Page DL and Anderson TJ (eds), pp. 300–311. Churchill Livingstone: Edinburgh

Gilchrist K, Kalsih L, Gould V, Hirschl S, Imbriglia JE, Levy WM, Patchefsky AS, Penner DW, Pickren J and Roth JA (1985) Interobserver reproducibility of histopathological features in stage 2 breast cancer. *Breast Cancer Res Treat* **5**: 3–10

Hayward JL, Rubens RD, Carbone PP, Henson JC, Kumaoka S and Segaloff A (1977) Assessment of response to therapy in advanced breast cancer. *Br J Cancer* **35**: 292–298

Heintz NH, Leslie KD, Rogers LA and Howard PL (1990) Amplification of the c-erbB-2 oncogene and prognosis in breast cancer. *Arch Pathol Lab Med* **114**: 160–163

Horak E, Smith K and Bromkuy L (1991) Mutant p53, EGF receptor and c-erb-B2 expression in human breast cancer. *Oncogene* **6**: 2277–2284

Isola J, Visakorpi T, Holli K and Kallioniemi OP (1992) Association of overexpression of tumor suppressor protein p53 with rapid cell proliferation and poor prognosis in node-negative breast cancer patients. *J Natl Cancer Inst* **84**: 1109–1114

Jacuerner J, Moles JP, Penault-Lorca F, Adelaine J, Torrente M, Vians P, Birngaum D and Theillet C (1994) p53 immunochemical analysis in breast cancer with four monoclonal antibodies: comparison of staining and PCR-SSCP results. *Br J Cancer* **69**: 846–852

Joensuu H, Toikkanen S and Isola J (1995) Stromal cell cathepsin-D expression and long term survival in breast cancer. *Br J Cancer* **71**: 155–159

Klijn JGM, Berns PMJJ, Bontenbal M, Alexieva-Figurch J and Foekens JA (1992) Clinical breast cancer, new developments in selection and endocrine treatment of patients *J Steroid Biochem Mol Biol* **43**: 211–221

Klijn JGM, Berns EMJJ, Bontenbal M and Foekens J (1993*a*) Cell biological factors associated with the response of breast cancer to systemic treatment. *Cancer Treatment Rev* **19** (suppl.B): 45–63

Klijn JGM, Berns EMJJ and Foekens JA (1993*b*) Prognostic factor and response to therapy in breast cancer. In *Cancer Surveys,* Vol. 18, Fentiman IS and Taylor-Papadimitriou J (eds), pp. 165–198. Coldspring Harbor Press: New York

Koechli OR, Schaer GN, Seifert B, Hornung R, Haller U, Eppenberger U and Mueller H (1994) Mutant p53 protein associated with chemosensitivity in breast cancer specimens. *Lancet* **344**: 1648–1649

Levine AJ, Perry ME, Chang A, Silver A, Dittmer D, Wu M and Welsh D (1994) The 1993 Walter Hubert Lecture: the role of the p53 tumour-suppressor gene in tumorigenesis. *Br J Cancer* **69**: 409–416

Makris A, Powles TJ, Dowsett M and Allred C (1995) p53 protein overexpression and chemosensitivity in breast cancer. *Lancet* **345**: 1181–1182

Marx J (1993) How p53 suppresses cell growth. *Science* **262**: 1644–1645

Mathieu M-C, Koscielny S, Le Bihan M-L, Spielmann M and Arriagada R (1995) p53 protein overexpression and chemosensitivity in breast cancer. *Lancet* **345**: 1182

McDivitt R, Stewart F and Berg J (1968) Tumours of the breast. In *Atlas of Tumor Pathology,* Firninger HJ (ed.), Armed Forces Institute of Pathology: Washington

Neubauer A, Neubauer B, He M, Effert P, Iglehart D, Furye RA and Liu E (1992) Technical report. Analysis of gene amplification in archival tissue by differential polymerase chain reaction. *Oncogene* **7**: 1019–1025

O'Donahue AEMA, Poller DN and Bell JA (1995) Cathepsin D in primary breast carcinoma: adverse prognosis is associated with expression of cathepsin D in stromal cells. *Breast Cancer Res Treat* **33**: 137–145

Ostrowski JL, Sarvan A, Henry L, Wright C, Henry JA, Hennessy C, Lennard TJ, Angus B and Horne CH (1991) p53 expression in human breast cancer related to survival and prognostic factors: an immunochemical study. *J Pathol* **164**: 75–81

Parkes HC, Lillyrop K, Howell A and Graig RK (1990) c-erbB-2 mRNA expression in human breast tumours: comparison with c-erbB-2 DNA amplification and correlation with prognosis. *Br J Cancer* **61**: 39–45

Poller DN, Hutchings CE, Galea MH, Bell JA, Nicholson RA, Elston CW, Blamey RW and Ellis IO (1992) p53 protein expression in human breast carcinoma: relationship to expression of epidermal growth factor receptor, c-erb-B2 protein overexpression and oestrogen receptor. *Br J Cancer* **66**: 583–588

Scarff R and Torlini H (1968) *Histological Typing of Breast Tumors*. WHO: Geneva

Slamon DJ, Clark GM, Wong SG, Levin WJ, Ullrich A and McGuire WL (1987) Human breast cancer: correlation of relapse and survival with amplification of the HER/neu oncogene. *Science* **235**: 177–182

Slamon DJ, Godolphin W, Jones LA, Holt JA, Wong SG, Kaith DE, Levin WJ, Stuart SG, Udove J and Ullrich A (1989) Studies of the HER-2/neu proto-oncogene in human breast and ovarian cancer. *Science* **244**: 707–712

Stenkvist B, Bengtsson E, Eriksson O, Jarksans T, Nordin B and Westman-Naeser S (1983) Histopathological grading system of breast cancer classification: reproducibility and clinical significance *J Clin Pathol* **36**: 392–398

© Cancer Research Campaign 1997 

Tandon AK, Clark GM, Chamness GC, Ullrch A and McGuire WL (1989) HER-2/neu oncogene protein and prognosis in breast cancer. *J Clin Oncol* **7**: 1120–1128

Tetu B, Brisson J, Coté C, Brisson S, Potvin D and Roberge N (1993) Prognostic significance of cathepsin D expression in node-positive breast carcinoma: an immunohistochemical study. *Int J Cancer* **55**: 429–435

Van de Vivjer MJ, Peterse JL, Mooi WJ, Wisman P, Lonans J, Dalesio O and Nusse R (1988) Neu-protein overexpression in breast cancer. *N Engl J Med* **319**: 1239–1245

Varley JM, Swallow JE, Brammer WJ, Whittaker JL and Walker RA (1987) Alterations to either c-erbB-2 (neu) or c-myc proto-oncogenes in breast carcinomas correlate with poor short term prognosis. *Oncogene* **1**: 432–430

Walker RA, Gullick WJ and Garley JM (1989) An evaluation of immunereactivity for c-erbB-2 protein as a marker of poor short-term prognosis in breast cancer. *Br J Cancer* **60**: 426–429

Walker RA, Dearing SJ, Lane DP and Varley JH (1991) Expression of p53 protein in infiltrating and in-situ breast carcinomas. *J Pathol* **165**: 203–211

Winstanley JHR, Leinster SJ, Cooke TG, Westley BR, Plats-Higgins AM and Rudland DL (1993) Prognostic significance of cathepsin-D in patients with breast cancer. *Br J Cancer* **67**: 767–772

Zhou DJ, Ahuja H and Cline MJ (1989) Proto-oncogene abnormalities in human breast cancer: c-erbB-2 amplification does not correlate with recurrence of disease. *Oncogene* **4**: 105–108

*© Cancer Research Campaign 1997*